# The Rehabilitation Journey of a Cricket Player With Partial Rotator Cuff Tear: A Case Report of Pre and Postoperative Physiotherapy

**DOI:** 10.7759/cureus.52336

**Published:** 2024-01-15

**Authors:** Shraddha S Kochar, Swapnil U Ramteke, Subrat Samal

**Affiliations:** 1 Sports Physiotherapy, Ravi Nair Physiotherapy College, Datta Meghe Institute of Higher Education and Research, Wardha, IND; 2 Musculoskeletal Physiotherapy, Ravi Nair Physiotherapy College, Datta Meghe Institute of Higher Education and Research, Wardha, IND

**Keywords:** case report, supraspinatus tendon tear, partial rotator cuff tear, preoperative rehabilitation, physiotherapy, rotator cuff tear

## Abstract

In adults, partial rotator cuff injuries can frequently be the root cause of pain in the shoulder. One recurrent pathology that may significantly impact a broad spectrum of individuals, including athletes, laborers, and sedentary people, is partial rotator cuff tears (RCTs). Physical therapy, anti-inflammatories, painkillers, rest or activity modifications, and corticosteroid injections are a few nonoperative treatment options for partial RCTs. We report a case of a 27-year-old male who sustained a rotator cuff injury of the right shoulder. The patient presented with pain and restriction of the right shoulder joint following the injury, which had occurred while throwing a ball forcefully with his right hand. The rehabilitation program emphasized pain management, restoring range of motion (ROM), increasing strength of muscles, and functional activities to optimize the patient's recovery. Concurrently, isometrics, ROM, and strengthening exercises were integrated into the rehabilitation program to enhance muscle strength, prevent tightness, and maintain ROM. The patient's progress was monitored by using the Disabilities of Arm, Shoulder, and Hand (DASH) score and the Upper Extremity Functional Scale (UEFS) at specific intervals during rehabilitation. The treatment and healing of a patient with an RCT who underwent both pre and postoperative physiotherapy are explored in this case report.

## Introduction

Rotator cuff tears (RCTs) constitute one of the most common causes of shoulder pain in adults, with an estimated incidence of 20-34% [1]. It has been demonstrated that rotator cuff injuries significantly affect functions in activities of daily living (ADLs) and induce significant pain and impairment [2]. The humeral head remains stabilized within the shoulder joint, and upper limb movement is facilitated by the four muscle-tendon units that make up the rotator cuff. When one or more of these units are entirely or partially interrupted due to a severe injury or degeneration, it is also known as an RCT [3]. Four muscles and tendons that surround the shoulder constitute the rotator cuff. These muscles are helpful in glenohumeral (GH) elevation and rotation, including the supraspinatus, infraspinatus, subscapularis, and teres minor [4]. Partial RCTs are highly prevalent and can cause difficulties in individuals. Several treatment options are available depending on the location and extent of the tear, as well as the individual features of each patient [5]. RCTs are typical in individuals who perform an overhead activity or repetitive lifting. RCTs associated with overuse are prevalent among athletes, especially in the setting of recurrent microtrauma [6].

Patients who report injuries to their rotator cuff might undergo conservative therapy or surgery. The first line of treatment is suggested to be conservative, involving physiotherapy, nonsteroidal anti-inflammatory drugs (NSAIDs), and intra-articular injections [7]. Among the multifunctional conservative treatment possibilities for RCTs are physical rehabilitation, modalities (such as heat therapy, electrotherapy, acupuncture, ultrasound, and ice therapy), taping, injection therapy, and medication administration [8]. Small to medium-sized injuries to the rotator cuff are frequently reported to respond effectively to both physical therapy and tendon restoration [9]. Physical therapy is vital to help patients recuperate from rotator cuff injuries since it promotes tendon healing after surgery. It ensures that shoulder strength and range of motion (ROM) have been recovered [10].

## Case presentation

Patient information

A 27-year-old male cricket player presented with pain and difficulty in movement of the right shoulder for 10 days, which had started while throwing a ball forcefully with his right hand. The pain was gradual in onset and increased progressively over 10 days, and increased with activities such as lifting heavy objects, reaching for something on a high shelf, and was relieved on rest. The patient had visited a local hospital and received medications for pain relief, but had not experienced any symptomatic relief. He subsequently presented to Acharya Vinobha Bhave Rural Hospital (AVBRH), where diagnostic investigations, including X-rays and MRI, were performed. Clinical diagnosis was done with a Full Can Test, which was positive and showed rotator cuff injury of the right shoulder. The MRI of the right shoulder subsequently revealed a partial tear of the supraspinatus tendon, and an X-ray showed a Hill-Sachs lesion in the posterosuperior aspect of the head of the humerus. On December 22, 2023, a surgical procedure involving soft tissue reconstruction for rotator cuff injury in the right shoulder was planned. The patient was referred to physiotherapy for seven days before the procedure. The primary postoperative symptoms of the patient were pain surrounding the shoulder joint and difficulty achieving a complete ROM. Physiotherapy was prescribed for additional care of these issues.

Clinical findings

On preoperative examination, the ROM of the right shoulder joint was significantly reduced, the Full Can Test was positive, and on palpation, grade III (patient winces and withdraws the affected part) tenderness was present around the right shoulder region. Upper limb manual muscle testing (MMT) could not be performed and ROM was not assessed due to pain preoperatively. After the operation, the patient reported dull, aching pain at the operated site, which aggravated movement, and was relieved on rest and medications. The pain was rated as 4.2/10 on the visual analog scale (VAS) on rest and 8.2/10 on movement, and experienced difficulty in shoulder movements and limitation in overhead mobility. During observation, a sling brace was present around the right shoulder joint. Upper limb MMT was done postoperatively, and the muscle strength was found to be reduced (Table 1). The active ROM of the shoulder joint was performed postoperatively, as shown in Table 2.

**Table 1:** Postoperative manual muscle testing of the right side 3-: performs movement against gravity greater than one-half range of motion (ROM); 4: performs full ROM against gravity with minimal resistance; 5: performs full ROM against gravity with maximal resistance

Joint	Muscles	Pre-rehabilitation (postoperative)	Post-rehabilitation (postoperative)
Shoulder	Flexors	3-/5	4/5
	Extensors	3-/5	4/5
	Abductors	3-/5	4/5
	Adductors	3-/5	4/5
	Internal rotators	3-/5	4/5
	External rotators	3-/5	4/5

**Table 2:** Postoperative active range of motion on the right side

Joint	Joint mobility	Pre-rehabilitation (postoperative)	Post-rehabilitation (postoperative)
Shoulder	Flexion	0^o^-30^o^	0^o^-155^o^
	Extension	0^o^-25^o^	0^o^-50^o^
	Abduction	0^o^-35^o ^	0^o^-145^o^
	Internal rotation	0^o^-25^o^	0^o^ -55^o^
	External rotation	0^o^ -35^o^	0^o^-60^o^

Investigation findings

**Figure 1:**
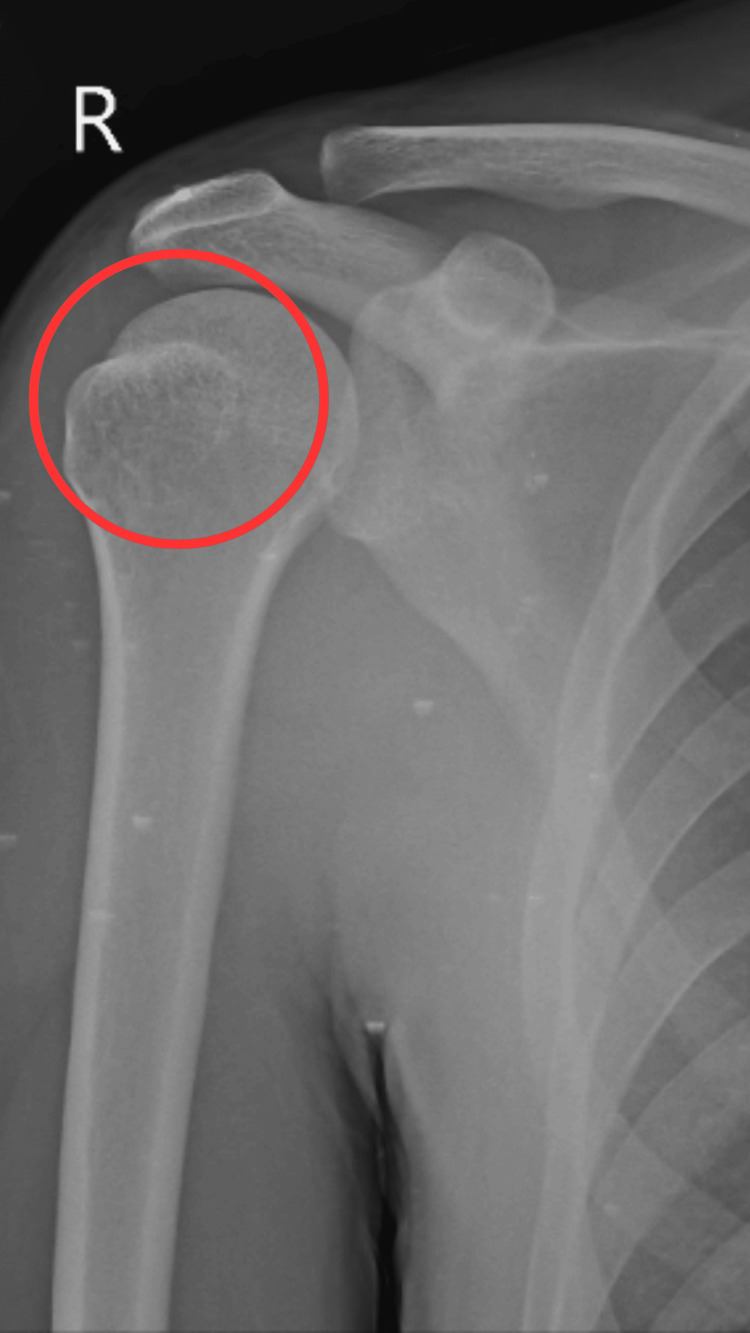
Preoperative X-ray of the right shoulder The red circle indicates a Hill-Sachs lesion in the posterosuperior aspect of the head of the humerus

Figure 1 shows the X-ray of the right shoulder joint preoperatively.

Therapeutic intervention

The patient underwent preoperative treatment for seven days, as described in Table 3. Physical therapy was subsequently continued following surgical intervention, as detailed in Table 4. Transcutaneous electrical nerve stimulation (TENS) was given to the patient for 10 minutes before starting the exercise program.

**Table 3:** Preoperative physiotherapy rehabilitation N/A: not applicable; reps: repetitions; PROM: passive range of motion; AROM: active range of motion

Goal	Treatment regimen	Dosage
Patient education	Patients and family members were educated about the injury, the significance of physical therapy, and how it will reduce to occurrence of secondary complications after the surgery	N/A
To reduce pain	Ice therapy	10-15 minutes for 3-4 times/day
To improve shoulder ROM	PROM exercises within pain limits which included forward flexion, internal and external rotation, as well as pendulum exercise	15 reps, 3 sets/day
To maintain the elbow and wrist joint ROM	Elbow flexion, extension exercises, and wrist AROM exercises	15 reps, 3 sets/day
To improve the strength of shoulder girdle musculature and scapula stabilizers	Isometric exercises without placing excessive stress on the affected site, and protraction retraction exercises for the scapula in prone and supine positions respectively. Isometric anterior deltoid contraction	15 reps, 5-second hold, 3 sets/day
To promote anterior and posterior capsule flexibility and minimize tightness	For the anterior capsule, door frame stretch was given; for the posterior capsule, cross-body stretch and towel stretch were given	30-second stretches, 5 sets/day

**Table 4:** Postoperative physiotherapy rehabilitation N/A: not applicable; QoL: quality of life; ROM: range of motion; PROM: passive range of motion; reps: repetitions; AAROM: active assisted range of motion; AROM: active range of motion; GH: glenohumeral

Goal	Treatment regimen	Dosage
Patient education	Patients and family members were educated about the posture that should be maintained, the importance of the brace, and how physiotherapy will help improve the QoL of the patient	N/A
To reduce pain	Ice therapy	20 minutes for 3-4 times/day
To maintain/protect the integrity of the repair	Use of abduction brace/sling (during sleep also), which was removed only during exercises	N/A
To improve joint ROM	PROM exercises were initiated initially to the shoulder joint, which progressed to AAROM and AROM exercises till the pain-free range. Shoulder pendulum exercises were started when the patient achieved AROM of the shoulder joint. Along with this, AROM exercises were given to the elbow, wrist, and fingers to maintain joint mobility. Shoulder shrugs were given	10 reps, 3 sets/day
To prevent muscular inhibition	Initially, scapular muscle isometrics were initiated. Gripping exercises for the fingers were also given. Later, to increase dynamic stabilization, supine GH submaximal rhythmic stabilization exercises were initiated	10 reps, 2 sets/day
To improve flexibility and reduce tightness of the anterior and posterior capsule	Internal rotator stretching was started. For the anterior capsule, door frame stretch was given; for the posterior capsule, cross-body stretch, towel stretch, and sleeper stretch were given	30-second stretches, 5 sets/day
Gradual return to functional activities	The patient was asked to perform sports-specific activities like putting on gloves and throwing the ball with medium intensity and then catching it; along with everyday activities such as putting on clothes and combing hair	10 reps, 3 sets/day

Outcome measures

The Disabilities of the Arm, Shoulder, and Hand (DASH) is a self-report questionnaire designed to assess disability and symptoms associated with upper limbs. The DASH score comprises 30 items that cover various aspects of symptoms, function, and social and psychological components related to the arm, shoulder, and hand. Subsequently, the total score is calculated and averaged, which amounts to 100, with higher scores reflecting a greater degree of upper extremity impairment and disability [11].

The Upper Extremity Functional Scale (UEFS) is a clinical evaluation tool to determine the functional status of the upper limbs, specifically the arms and hands. It is frequently used to assess the functional abilities of individuals with neurological or musculoskeletal conditions that affect the upper limbs in rehabilitation settings. It consists of 20 items [12]. Table 5 shows pre and postoperative outcome measures in our patient as per DASH and UEFS.

**Table 5:** Outcome measures VAS: visual analog scale; DASH: Disabilities of the Arm, Shoulder, and Hand; UEFS: Upper Extremity Functional Scale; N/A: not assessable

Outcome measures	Preoperative		Postoperative	
	Pre-rehabilitation	Post-rehabilitation	Pre-rehabilitation	Post-rehabilitation
VAS	On rest: 4.5/10, on movement: 8.3/10	On rest: 3.8/10, on movement: 6.7/10	On rest: 4.2/10, on movement: 8.2/10	On rest: 2.5/10, on movement: 4.1/10
DASH score	N/A	N/A	N/A	65/100
UEFS	6/80	10/80	36/80	68/80

## Discussion

We discussed a case of a 27-year-old male patient, a cricketer who sustained an injury while throwing a ball in high intensity. The patient was diagnosed with an RCT clinically, and an MRI suggested a partial tear of the supraspinatus tendon of the right shoulder. For this, the patient was referred to preoperative physical therapy to reduce the occurrence of secondary complications after the surgical procedure. After the operation, postoperative rehabilitation was performed. Both the pre and post-physiotherapy rehabilitation courses were found to be clinically significant. The pre-and post-outcome measures assessment found that the patient’s QoL improved significantly following the therapy.

A study by Edwards et al. showed that physiotherapy was effective for the nonoperative management of rotator cuff injury. It was crucial to recognize that the probability of a successful exercise rehabilitation program depended on the individual’s responsiveness and symptom recurrence. The rehabilitation protocol in this study included pendulum exercises that improved the ROM of the shoulder joint, door frame stretches, and towel stretches that increased the strength of the muscle, and scapular protraction and retraction exercises that enhanced the strength of the scapula stabilizers [13]. These exercises were implemented in our patient as well and were found to be effective in increasing ROM, muscular strength, and flexibility. As suggested by Dickinson et al., the evidence in the literature indicates that cryotherapy or ice therapy may be beneficial for pain management, sleep quality, medication reduction, and improving participation in therapeutic exercises. An ice pack wrapped in an ACE bandage can be more affordable and is as efficient as advanced continuous cryotherapy facilities [14]. Cryotherapy was also implemented and found to be effective. Research by Conti et al. has reported that postoperative physiotherapy plays a significant role in managing secondary complications and improving patients' ADLs, thereby enhancing their QoL [15].

## Conclusions

For patients with partial RCT, an effective rehabilitation regimen that involves both preoperative and postoperative care is crucial for favorable long-term recovery. This tear often remains unnoticed, but it should be addressed as soon as severe symptoms begin, causing issues with everyday tasks and QoL. This case report described a cricket player who suffered a partial RCT and underwent soft tissue reconstruction for his right shoulder rotator cuff injury and was referred to the physiotherapy department for both pre and postoperative rehabilitation. He received physiotherapy treatment that was tailored for him. The patient reported enhanced ROM, strength in the muscles surrounding the shoulder, functional independence, ADL, as well as increased UEFS. We believe this case report contributes to the body of knowledge on the preoperative management of these patients, especially given the scarce data on preoperative treatment in the current literature.
